# Mindfulness-based stress reduction is linked with an improved Cognitive Reflection Test score

**DOI:** 10.3389/fpsyg.2023.1272324

**Published:** 2023-10-03

**Authors:** Léa Lachaud, Baptiste Jacquet, Maxime Bourlier, Jean Baratgin

**Affiliations:** ^1^Université Paris 8 (UP8), Laboratoire Cognitions Humaine et Artificielle (CHArt), Saint-Denis, France; ^2^Université de Paris-Est Créteil, Laboratoire CHArt-UPEC, Créteil, France; ^3^Probability, Assessment, Reasoning and Inferences Studies (P-A-R-I-S) Association, Paris, France

**Keywords:** mindfulness, MBSR, CRT, dual-process, intuitive thinking

## Abstract

Initially, dual-process theories suggested that the existence of two different cognitive systems explained why many participants do not find the correct answer in many reasoning tasks. The Cognitive Reflection Test (CRT) is one such task. It contains three questions with incorrect answers (typically associated with intuition and thus system 1 which processes information automatically) and correct answers (typically associated with deliberate thinking and thus system 2 which involves the conscious processing of information). More recent theories suggest system 1 is responsible for both incorrect *and* correct responses, with system 2 being used to resolve the conflict between these different intuitions. Since mindfulness training improves self-regulation and cognitive flexibility, we believe it could improve CRT scores by reducing the relative weight of initial intuitions by strengthening alternative intuitions, thus increasing the probability of triggering deliberate reasoning. To test this hypothesis, we recruited 36 participants, all registered in the same Mindfulness-Based Stress Reduction (MBSR) training. Of those 36 participants, 18 answered the CRT before the training and 18 answered it after 8 weeks of training. Results show that participants who followed MBSR training had better CRT scores than those without training. This is coherent with our hypothesis that mindfulness training could reduce the relative weight of initial intuitions and facilitate deliberate thinking.

## 1. Introduction

Mathematical riddles are popular because they present the challenge of not falling into the traps that they set. These traps, or lures, exist because the mind functions on the basis of a reasoning system that sometimes generates cognitive biases. It is these biases that lead reasoners to incorrect conclusions in math puzzles, but they are also what makes them interesting and fun.

For example, the “lily pads” riddle sets the following trap: *In a lake, there is a patch of lily pads. Every day, the patch doubles in size. If it takes 48 days for the patch to cover the entire lake, how long would it take for the patch to cover half of the lake?* Two answers are usually generated by reasoners (Frederick, 2005). The first answer, *24 days*, is quick and intuitive, and the correct answer, *47 days*, is slower to obtain. The trap set by the riddle is that of the intuitive and quick answer. Although most reasoners first think of the answer *24 days*, the correct answer turns out to be *47 days*. The initial intuition suggests that we should divide 48 by 2 because the puzzle indicates that the lily pads are doubling in size, when in fact we should take into account the fact that the lily pads are growing exponentially.

This puzzle has been integrated into the Cognitive Reflection Test (CRT; Frederick, 2005) which is used to study this switch between the generation of the false intuitive answer and the true deliberate answer. The CRT consists of three riddles of a similar style (Table 1).

**Table 1:** The three riddles of the Cognitive Reflection Test (CRT).

**Riddle name**	**Riddle**	**Answers**	
		**Intuitive (false)**	**Deliberate (correct)**
(1) The bat and ball	A bat and a ball cost $1.10 in total. The bat costs $1.00 more than the ball. How much does the ball cost?	10 cents	5 cents
(2) The machines	If it takes five machines 5 min to make five widgets, how long would it take 100 machines to make 100 widgets?	100 min	5 min
(3) The lily pads	In a lake, there is a patch of lily pads. Every day, the patch doubles in size. If it takes 48 days for the patch to cover the entire lake, how long would it take for the patch to cover half of the lake?	24 days	47 days

Studies have suggested that participants who did not fall into the trap of intuitive response were more likely to adopt an analytical mode of thinking (Frederick, 2005; Brañas-Garza et al., 2019). This mode is classically attributed to system 2, which allows reasoners to deliberate in order to generate a non-automatic or non-intuitive response (Stanovich and West, 2000; Kahneman, 2003, 2011). Various studies have shown that mindfulness appears to be a facilitator of de-automatization (Wenk-Sormaz, 2005; Chan and Woollacott, 2007; Moore and Malinowski, 2009; Lachaud et al., 2022). It could be so that mindfulness can be an aid in generating the right answer to this type of puzzle.

### 1.1. Intuitive thinking makes you fail in the Cognitive Reflection Test

The CRT is based on traditional dual information processing theories (Frederick, 2005). These theories state that there are two cognitive systems for processing information: the automatic mode and the controlled mode. The first corresponds to what is also called system 1. It is a reasoning system that has the properties of (i) being uncontrolled, (ii) being effortless, (iii) being associative, (iv) being fast, (v) being unconscious, and (vi) being intuitive. It allows for instantaneous problem-solving and decision-making through heuristics. In contrast, the second is associated with the effortful and deductive system 2. It has the properties of (i) being non-automatic, (ii) requiring effort, (iii) being deductive, (iv) being slow, (v) being conscious, and (vi) applying rules (Kahneman, 2011).

Traditional theories of dual information processing (Stanovich and West, 2000; Kahneman, 2003) assume the exclusivity of the two systems, which means that the intuitive response is always generated by the automatic mode (system 1) while the deliberate response is always generated by the controlled mode (system 2). The issue is then to understand how reasoners manage to change from the intuitive response generated exclusively by system 1 to the deliberate response generated exclusively by system 2. The explanation used in this theory is that there is a trigger that functions like a switch allowing the reasoner to switch from one system to the other. To do this, system 2 would continuously monitor the results of system 1, which would then be activated in case of conflict between the two systems (Stanovich and West, 2000; Kahneman, 2011). This monitoring is considered to be lax because it allows the generation of (sometimes) erroneous intuitive judgments (Kahneman and Frederick, 2002). This laxity could explain the high failure rate at the CRT.

However, De Neys (2022) and Evans (2019) point to a paradox in the explanation of how the switch between the two systems would work. If it is indeed system 2 that detects the conflict between the responses of system 1 and system 2, this implies that system 2 must be engaged first to calculate its own response. In response to this paradox, a more recent alternative theory suggests that system 1 would trigger the deliberation performed by system 2 (Pennycook et al., 2015; De Neys, 2022). This trigger would then be of an intuitive nature because the monitoring of system 1 is no longer attributed to system 2 but to system 1 itself. In this theory, the monitoring system is explained by the uncertainty generated by two intuitive and contradictory responses. More precisely, different processes belonging to system 1 could produce two responses of an intuitive nature in parallel: the response traditionally attributed to system 1, and an alternative response traditionally attributed to system 2 (De Neys and Glumicic, 2008). If these two responses, both intuitive in nature, generate the same answer, or if one of the two is much stronger than the other, the deliberative system 2 is not triggered, and the reasoner remains in a default type I reasoning mode. Otherwise, if two sufficiently strong contradictory responses are generated, system 2 is triggered to try to resolve the conflict and consciously generate the response.

Since system 2 is triggered due to a conflict between intuitions, we suppose its use would be promoted by strengthening alternative intuitions. Indeed, strengthening them would result in a decreased immediacy of the incorrect initial response by reducing the difference of strength between the two intuitions.

### 1.2. Mindfulness training as a potential tool to reduce the relative weight of initial intuitions

We believe the practice of mindfulness could increase the strength of alternative intuitions and, in doing so, reduce the relative weight of the intuition leading to wrong answers in the CRT. Mindfulness is a natural predisposition trained and maintained by meditative practices (Brown and Ryan, 2003). It aims at improving self-regulation by developing attentional control, emotional regulation, and self-awareness (Malinowski, 2013; Tang et al., 2015). The repetition of mindfulness training would allow learning, developing, and automatizing the ability to switch more quickly from one piece of information to another. During the practice of a focused attention meditation, the meditator is led to perform *mental set shifting* through five activities during which they exercise a constant shifting of their mental states (Miyake et al., 2000; Malinowski, 2013):

(a) The sustained attention process: The meditator begins their meditation by focusing their attention on a particular object. In this mental state, type II processes are activated : They are controlled or deliberate, and close to metacognition because the meditator observes their own cognitive functions.
(b) The distraction process: In this second stage, the meditator will naturally be carried away by their own thoughts in a state of mind-wandering based on type I processes, i.e., automatic and uncontrolled.
(c) The monitoring process: During this third stage the meditator recognizes that they are in a state of mind-wandering and thus are able to consciously observe this process. They essentially take a step back, “detach themselves,” from the automatic aspect of the mind-wandering that just occurred.
(d) The disengagement process: Once they have detached themselves, the next step is to abandon the mind-wandering.
(e) The attentional shift process: Finally, the meditator returns to the original object by shifting their attention back to it.

Therefore, mindfulness practice uses monitoring functions and consists of the constant adjustment of one's attentional focus (Malinowski, 2013). This could help modulate the strength of intuitions through a process of observation that would reduce the degree of automatization (De Neys, 2022). Thus, the challenge of focused attention meditation is to take control back whenever automatic processes distract us. Through regular repetition of this type of training, the practice of mindfulness could in some way allow for the *automatization of de-automatization*. Several studies have already suggested that mindfulness facilitates cognitive de-automatization in certain experimental situations (Wenk-Sormaz, 2005; Chan and Woollacott, 2007; Moore and Malinowski, 2009; Lachaud et al., 2022). Furthermore, mindfulness training has also been shown to reduce the *Einstellung effect* (Greenberg et al., 2012) (i.e., being unable to find a better solution when one already seems to be working; Luchins, 1942), another piece of evidence of cognitive de-automatization through the practice of mindfulness. This suggests that mindfulness could increase CRT scores by generating more deliberate responses.

### 1.3. Does mindfulness training improve scores in the Cognitive Reflection Test?

The purpose of this study is to verify if mindfulness training improves the CRT score. Farrar et al. (2020) have already studied the influence of brief exposure to a mindfulness exercise and of the trait mindfulness on the score in a variant of the CRT (Toplak et al., 2014). No influence of this exposure on the rate of correct responses was observed. One interpretation the authors make is that the mindfulness exercise may have been too brief to significantly promote one's ability to detach themselves from automatic processes (i.e., what Evans and Stanovich, 2013 calls *cognitive decoupling* in the context of reasoning). In addition, a systematic review of the influence of mindfulness on cognition showed that several regular sessions of mindfulness were necessary to obtain an effect (Chiesa et al., 2011; Khoury et al., 2017). This difference between short exposure and longer exposure could be the result of a lack of expertise in the process of de-automatization. We believe that comparing CRT performance between mindfulness-trained and untrained participants could provide a better understanding of this cognitive decoupling skill.

The time and frequency of mindfulness training can be controlled through participation in instructor-led training aimed at practicing mindfulness every day for several weeks. Mindfulness-Based Stress Reduction (MBSR) is one such training that should be relevant due to practicing exercises focused on decoupling using *mental set shifting* (Kabat-Zinn, 1990).^1^ We, therefore, expect MBSR-trained participants to have more experience in de-automatization compared to untrained participants, and consequently to score higher on the CRT.

It is important to also consider control factors such as the period of the MBSR training (round), the attention paid to the experiment reported by participants, and the gender of the participants as they could potentially influence the CRT score (Oldrati et al., 2016; Ring et al., 2016; Brañas-Garza et al., 2019). We also controlled age, which could influence the trait mindfulness (Vujic, 2017). The impact of the trait mindfulness itself on the CRT has been studied before but no direct relation could be found (Farrar et al., 2020). We included trait mindfulness to verify this observation. As the prior meditation practice could also have an effect on the CRT (for similar reasons that the MBSR training could have an effect), a measure of the experience in meditation should also be included in our analyses. Finally, given that regular mindfulness practice increases the frequency of the phenomenon of *Fringe consciousness*^2^ (Norman, 2017), we suspect that participants following MBSR training who would fail to answer the CRT question correctly would report lower confidence in their answers compared to participants without this training in the same situation. This would be caused by competing intuitions.

## 2. Method

### 2.1. Participants

Greenberg et al. (2012) reported that a sample size of 35 participants was required to observe a large effect size in a comparison between a mindfulness-trained group and a control group. Thus, 36 participants were recruited to participate in an 8-week MBSR training (11 males and 25 females, $\bar{Age}=43$; *SD* = 9.8). Participants were recruited from among the clients of a meditation instructor authorized to teach the MBSR program by the Association for the Development of Mindfulness (ADM, 2009). None of the participants had taken part in the MBSR program before starting the experiment. All of them were interested in mindfulness, were native French speakers, and did not know the CRT.

Given the small groups (from 8 to 10 participants) in the training programs of this instructor, two rounds of recruiting were carried out, each containing two sessions. Similarly to Greenberg et al. (2012), participants in each round were randomly assigned to either the first training session, corresponding to the post-MBSR condition ($N=10,\bar{Age}=44.9,SD=7.2$, and $N=8,\bar{Age}=44.3,SD=13.3$) or to a waiting list for the second training session, corresponding to the pre-MBSR condition ($N=10,\bar{Age}=42.8,SD=10.3$, and $N=8,\bar{Age}=40.4,SD=9.4$). Thus, the pre-MBSR group was composed of participants who were on the waiting list at the time of taking the CRT (14 females and 4 males), while the post-MBSR group was composed of participants who took the CRT after the 8 weeks of training (11 females and 7 males).^3^ The training received in each session consisted of the same program and was delivered by the same instructor in the same methods.

The experiment was carried out in accordance with the Helsinki Declaration. All participants gave their written informed consent to participate freely and anonymously in the study, with no financial compensation. They could stop the experiment at any moment and were debriefed at the end of the experiment.^4^

### 2.2. Material and procedure

For the post-MBSR condition, the training consisted of 2 h online (via ZOOM software) per week, accompanied by an instructor, to learn meditative techniques and to exchange around this practice. Participants were committed to performing mindfulness exercises for the duration of the MBSR training (a minimum of 15 min a day). The instructor had indeed underlined the importance of respecting this instruction in order to reap the benefits of this training.

We designed our questionnaire on SoSci Survey in French. We used standard CRT consisting of three riddles, available in Table 1 (Frederick, 2005).

The Freiburg Mindfulness Inventory (*FMI-14* items) was used (Walach et al., 2006) in its validated French version (Trousselard et al., 2010) to measure the trait mindfulness.

All participants completed the tests on their computers. Participants in the pre-MBSR condition performed the tests during their first appointment with the mindfulness instructor, just before starting the MBSR program. In contrast, participants in the post-MBSR condition completed it at the end of the last meeting of the training. The experiment began with the CRT (standard order: “bat and ball,” “machines,” and “lily pads”). After this, participants were asked to indicate on two 5-point scales how attentive they had been during the experiment and to what extent they thought they had given the correct answer to each question. The experiment ended with the FMI and then the socio-demographics questionnaire. Participants were asked to indicate whether they were already practicing meditation before taking part in the MBSR program (*yes* or *no*). If yes, they were asked to indicate for how long (this duration included the MBSR training). Participants were given unlimited time to answer the questions.

### 2.3. Data analysis

We used regression models to analyze our data which were then compared according to three criteria: the AIC (Akaike Information Criterion), which tends to prefer too complex models; the BIC (Bayesian Information Criterion), which tends to prefer too simple models (Kuha, 2004, for a comparison of AIC and BIC); and the Bayes Factor (BF) with a unit prior, calculated using BIC. For the Bayes Factor, a higher value indicates more evidence of one of the models. For AIC and BIC, lower values indicate a better model. All models were of the logistic family to take into account success rates on three questions.

## 3. Results

We first checked if the post-MBSR group obtained higher FMI scores than the pre-MBSR group. To do this, we used two alternative models to explain FMI scores.^5^ One included participation in the training as a factor (H1), and the other was without any explanatory factor (H0). The model with the group as a factor was better on all four criteria (H1: AIC = 249, BIC = 252; H0: AIC = 257, BIC = 259; *BF*_1*A*, 0*A*_ = 24, see Table 2) with the post-MBSR condition having higher FMI scores (*M* = 35.8, *SE* = 1.45) than the pre-MBSR condition (*M* = 29.8, *SE* = 1.05). Additionally, there was no effect of the session on FMI scores.

**Table 2:** Summary of the results of the models for the group validation.

**FMI as dependent variable**			
**Model name**	**AIC**	**BIC**	*BF*_1*A*, 0*A*_
**Group validation**			
Model 0A (Null)	257	259	
Model 1A (MBSR)	249^⋆^	252^⋆^	24
**Control factors**			
**Model name**	**AIC**	**BIC**	*BF*_2*A*, 1*A*_
Model 2A (Round)	259	262	0.010
**Prior practice as dependent variable**			
**Model name**	**AIC**	**BIC**	*BF*_1*B*, 0*B*_
**Group validation**			
Model 0B (Null)	122	125^⋆^	
Model 1B (MBSR)	121^⋆^	126	0.754
**Control factors**			
Model name	AIC	BIC	*BF*_2*B*, 0*B*_
Model 2B (Round)	124	129	0.168

⋆ Best model. For FMI, the AIC and BIC both agree that MBSR training is an important predictor of FMI scores. For prior meditation practice, AIC favors a difference depending on MBSR training while the BIC instead favors independence of MBSR training.

*BF*_1*A*, 0*A*_: Bayes Factor indicates how much better model 1A is compared to model 0A. A value of 24 indicates that model 1A is 24 times more likely to explain our data than model 0A.

*BF*_2*A*, 1*A*_: Bayes Factor indicates how much better model 2A is compared to model 1A. A value of 0.01 indicates that model 2A is 100 times less likely to explain our data than model 1A.

*BF*_1*B*, 0*B*_: Bayes Factor indicates how much better model 1B is compared to model 0B. A value of 0.754 indicates that model 1B is 1.3 times less likely to explain our data than model 0B.

*BF*_2*B*, 1*B*_: Bayes Factor indicates how much better model 2B is compared to model 1B. A value of 0.168 indicates that model 2B is 6 times less likely to explain our data than model 1B.

Additionally, we also created models to confirm that our groups were not different on their prior practice of meditation. The results show an ambiguity, with the AIC favoring the model predicting that the Pre-MBSR and Post-MBSR groups had different prior meditation practice, while the BIC instead favored the model showing no difference between the two group (H1: 121, BIC: 126; H0: AIC = 122, BIC = 125; *BF*_1*B*, 0*B*_ = 0.754). As a result, we include this potential factor in the models attempting to predict CRT scores.

**Figure 1:**
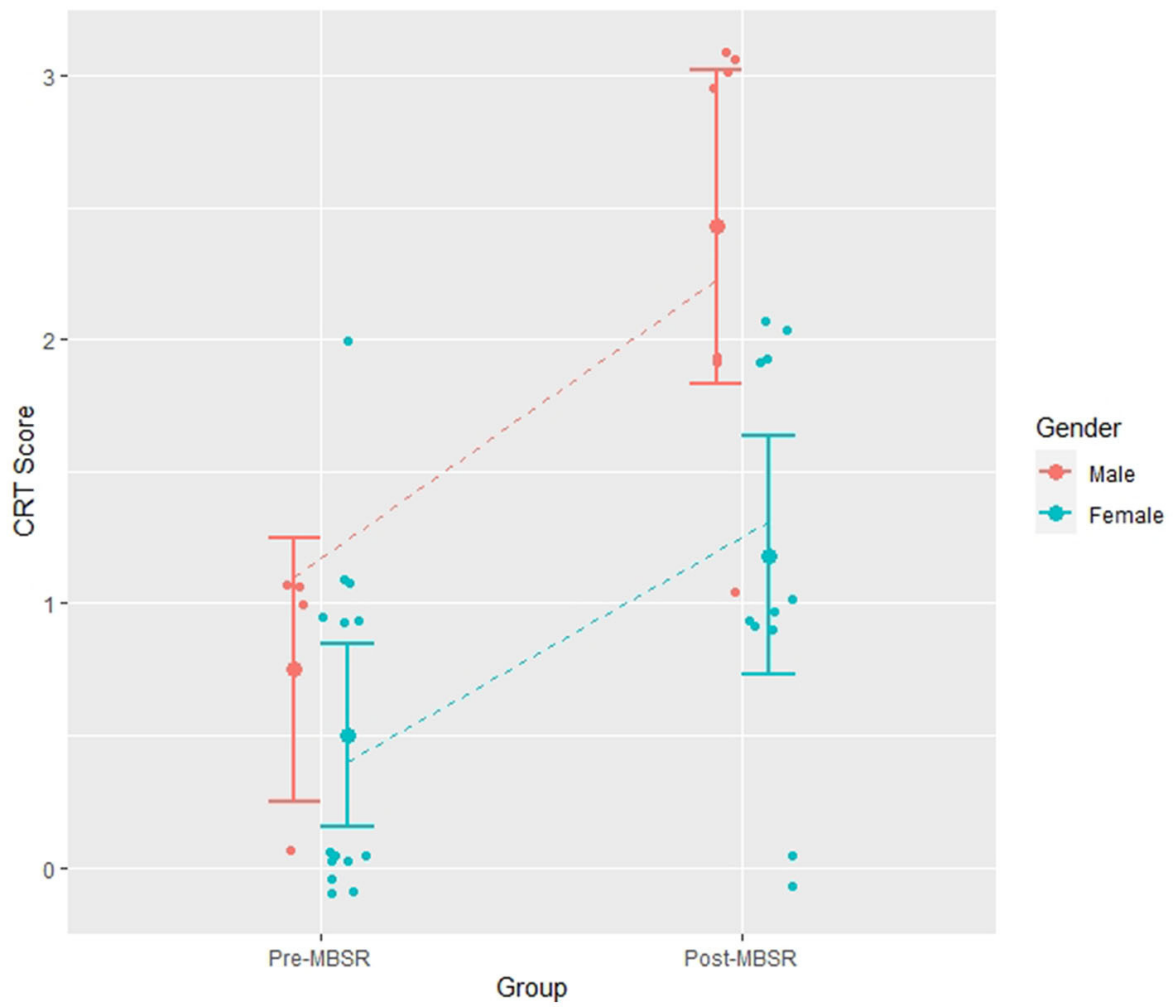
CRT Score depending on MBSR Training. The post-MBSR condition had higher CRT scores than the pre-MBSR condition, and males had higher scores than females. The error bars indicate the standard error and the dashed lines indicate the predictions of model 7 (MBSR+Gender), *N* = 36.

The comparison of the models with the CRT scores as a dependent variable showed a significant influence of the condition (pre-MBSR vs. post-MBSR) on the CRT scores with an additional effect of the gender of participants and a potential influence of their attention level [Model 11 (MBSR+Attention+Gender): AIC = 76, BIC = 83; Model 1 (MBSR): AIC = 84, BIC = 87; *BF*_11,1_ = 8.7], with the AIC being better for Model 11 (MBSR+Attention+Gender), but the BIC being slightly better for Model 7, which only includes the effect of the condition and of gender on CRT scores [Model 7 (MBSR+Gender): AIC = 78, BIC = 83; Model 1 (MBSR): AIC = 84, BIC = 87; *BF*_7,1_ = 9.2]. See Table 3 for a comparison of the models. A graphical representation of participants' answers and Model 7 predictions are shown in Figure 1.

**Table 3:** Summary of the results of the main hypothesis testing.

**Model name**	**AIC**	**BIC**	***BF*_10_**
**Main hypothesis testing**			
Model 0 (Null)	98	99	
Model 1 (MBSR)	84	87	618
**Control factors**			
**Model name**	**AIC**	**BIC**	*BF*_*X*1_
**FMI**			
Model 2 (FMI)	100	103	0.0
Model 3 (MBSR*FMI)	86	92	0.08
**Age**			
Model 4 (Age)	100	103	0.0
Model 5 (MBSR*Age)	87	93	0.04
**Gender**			
Model 6 (Gender)	89	92	0.07
Model 7 (MBSR+Gender)	78	83^⋆^	9.2
Model 8 (MBSR*Gender)	78	84	3.9
**Attention**			
Model 9 (Attention)	87	91	0.2
Model 10 (MBSR+Attention)	80	85	2.6
Model 11 (MBSR+Attention+Gender)	76^⋆^	83	8.7
**Round**			
Model 12 (Round)	100	103	0.0
Model 13 (MBSR+Round)	85	90	0.2
Model 14 (MBSR*Round)	87	93	0.04
**Practice**			
Model 15 (Practice)	95	98	0.003
Model 16 (MBSR+Practice)	85	90	0.26
Model 17 (MBSR+Practice+Gender)	79	85	2.35
Model 18 (MBSR+Practice*Gender)	79	87	0.90

^+^Additional effect.

^*^Interaction effect.

⋆ Best model: The AIC favors the model with additional effects of MBSR training, Attention, and Gender. The BIC favors the model containing only MBSR training and Gender as relevant predictors of CRT scores.

*BF*_10_: Bayes Factor indicating that model 1 is 618 times as good as model 0 to explain our data.

*BF*_*X*1_: Bayes Factor indicating how much better the given model (X from 2 to 14) was compared to the best model (model 1). For example, model 7 is 9 times as good as model 1 to explain our data as *BF*_71_ = 9.2.

MBSR, Mindfulness-Based Stress Reduction; FMI, Freiburg Mindfulness Inventory; Attention, Level of attention throughout the experience; Round, MBSR training periods; Practice, Self-reported practice of meditation.

The best model in terms of AIC (Model 11: MBSR+Attention+Gender) indicates that participants who followed MBSR training had higher CRT scores (2.16 times higher) than those who had not followed it, that males had higher CRT scores (1.72 times higher) than females in general, and that participants with a higher self-reported attention score had better CRT scores compared to those with low attention scores (each additional point of attention increasing the score by a factor of 1.26). The best model in terms of BIC (Model 7: MBSR + Gender) instead indicates that attention level had no influence on CRT Scores.

It is worthy of note that none of the models containing self-reported prior practice of meditation came up in the best models. Indeed, Model 17 which included MBSR training, prior practice and gender was worse than Model 11 and Model 7 on AIC and BIC [Model 17 (MBSR+Practice+Gender): AIC = 79, BIC = 85; Model 7 (MBSR+Gender): AIC = 78, BIC = 83; Model 11 (MBSR+Attention+Gender): AIC = 76, BIC = 83]. Thus, prior practice was not a good predictor of CRT Scores.

Since the CRT items each tackle different concepts (Bar-Hillel et al., 2018), we also investigated whether this effect could be found between the individual riddles of the CRT, following traditional practice in the literature (Brañas-Garza et al., 2019). A main effect of the condition (Pre-MBSR or Post-MBSR) was found for the “bat and ball” riddle (*Z* = −2.278, *p* < 0.05, with 6% correct answers in pre-MBSR condition compared to 50% in the post-MBSR condition) and for the “machines” riddle (*Z* = −2.208, *p* < 0.05, with 11% correct answers in pre-MBSR condition compared to 61% in the post-MBSR condition) but not for the “lily pads” riddle (39% compared to 44%). On the other hand, the “lily pads” riddle was the only one to show a significant influence of gender on the score (*Z* = −2.272, *p* < 0.05, with 82% of correct answers for males compared to 32% for females).

The self-reported confidence rate for each riddle was predicted by the self-reported level of attention in two riddles (“bat and ball” and “machines”) with a positive correlation between the two. For the “lily pads” riddle the self-reported confidence rate was predicted by whether the participant had correctly responded (a correct answer being correlated with a higher confidence rating).^6^

## 4. Discussion

This study showed that CRT scores could be improved by mindfulness training. The post-MBSR condition achieved higher CRT scores than the pre-MBSR condition, especially for males. Our results suggest that long and regular exposure to mindfulness would increase the likelihood of providing correct responses to the CRT, thereby lowering the likelihood of our initial intuition causing incorrect responses.

When the riddles are considered one by one, we note that the improvement in the rate of correct answers to the “bat and ball” and “machines” questions is influenced by MBSR. However, this training did not improve the score of the “lily pads” question. In addition, just as we observe in our study, the “lily pads” riddle is more often correctly answered overall compared with the other two, even more so among men. This result is difficult to interpret, but it could be due to the fact that this question is less cognitively demanding compared to the other two. As the baseline level of correct responses is already high, mindfulness could have a less prominent effect.

Our results are coherent with our hypothesis that mindfulness training could allow participants to switch more easily from intuitive system 1 to deliberate system 2 through the process of cognitive decoupling. Cognitive decoupling is considered to be a characteristic of Type II processes and is characterized by the ability to distinguish between assumptions and beliefs (Evans and Stanovich, 2013). Cultivating mindfulness could promote this decoupling through four specific mechanisms: awareness, attention, focus on the present, and acceptance (Kang et al., 2013). The mindfulness-trained group in our study was able to train the capacity for cognitive decoupling through the exercise of *mental set shifting* (Miyake et al., 2000). While a similar effect was not observed in Farrar et al. (2020), the reason might be that the authors only used a short mindfulness induction (15 min) instead of a longer mindfulness training.

The state of mindfulness could be a proactive behavior that increases attentional flexibility through exercises consisting of focusing attention on continually changing information (Kang et al., 2013). This flexible allocation of attentional resources could improve the detection of contextual cues present in the CRT that trigger an adaptation of reasoning strategies. The capacity for cognitive decoupling would make it possible to improve conscious access to additional cues that are necessary to solve the riddle. From the point of view of the current theory (De Neys, 2022), the perception of more task-relevant cues could reinforce alternative intuitions, thereby increasing the uncertainty of our initial intuition beyond a threshold, and in consequence trigger system 2. This is coherent with the observation that, in “lily pads” riddle, participants who gave the wrong answers had lower confidence in those answers compared to participants who gave the correct answers. However, the “bat and ball” and “machines” riddles do not follow this pattern because the confidence score is just as high whether the answers given are correct or incorrect. This may be due to the fact that, in these riddles in particular, the intuitive (and wrong) response generates a strong impression of rightness, just as strong as that given by the answer emerging from deliberate reasoning. This was not the case in the “lily pads” in which the confidence for wrong responses was lower. Perhaps in the “bat and ball” riddle, the initial intuition is so strong because participants immediately think about having to do a simple subtraction and feel quite confident in their ability to calculate its result, not realizing that it is the wrong operation to be applied in this context. Similarly, in the “machines” riddle answering “100” is quite a strong intuition due to two processes: the immediate analogy that can be done from the repetitive structure of the sentence and the desire to proportionally increase (multiplying all quantities by 20) the number of minutes.

It is possible that the decline in confidence observed when the answer was incorrect in the “lily pads” riddle would result from the alternative intuitions being present but not strong enough to emerge consciously as would be the case for a *Fringe Consciousness* effect (Mangan, 2001). This phenomenon may be a characteristic of mindfulness and regular practice could increase its frequency and become generalized to other tasks over the long term (Norman, 2017). Yet, should this be true, we should observe lower confidence when participants gave a wrong answer in the post-MBSR condition compared to that of the pre-MBSR condition.

This is not what we observed. Since post-MBSR participants had a higher likelihood of giving correct answers, it is possible that they were able to focus on the reason why they had this feeling of wrongness. In doing so, they might have strengthened the alternative intuitions enough to bring the correct answer to a conscious level and select it as their answer. In consequence, our results do not necessarily show a lack of *Fringe Consciousness* for the post-MBSR condition, but simply that, if it existed, it was already too late to observe it when they gave their final answer.

While the results showed an effect of MBSR on the CRT score, this was not the case for the mindfulness trait measured by the FMI. Although the FMI score was higher in the post-MBSR condition compared to the pre-MBSR condition, it did not predict high CRT scores. Studies have indeed shown that the FMI is not always understood in the same way among meditators and non-meditators (Belzer et al., 2013) due to the Dunning-Kruger effect (Kruger and Dunning, 1999; Dunning, 2011), with non-experts rating themselves higher than they should and experts rating themselves lower than they should. Thus, FMI could be measuring something different from the *automatization of de-automatization*, which only regular training could provide.

Our results also indicate an effect of gender and a potential effect of attention on CRT scores. To the best of our knowledge, no study has investigated the impact of attention during problem-solving on CRT scores (although the reverse has been studied in Welsh, 2022). We believe this link could be interesting to study in the context of mindfulness practice. Our results are in part consistent with previous studies (Frederick, 2005; Ring et al., 2016; Brañas-Garza et al., 2019; Otero and Alonso, 2023) regarding a gender effect. We observed a gender effect on the overall CRT score, but further investigation of each riddle only showed this effect on the “lily pads” riddle. This differs from the literature, in which gender effects are commonly observed with all three riddles. Regardless, mindfulness training similarly increased these scores for all genders.

It would be interesting to replicate this study with more participants while using an active control group to strengthen these results. The design of the study could be improved to assess the effect of the evolution of mindfulness training on the ability to trigger the deliberative system. The use of the FMI scale (chosen because it is quick to administer) could also be replaced by a longer but more complete scale (Baer et al., 2008). Finally, we could explore the possible occurrence of undesirable effects during online therapeutic programs based on mindfulness (Britton et al., 2021).

The CRT is used as an indicator of this ability to switch. However, it can also be understood as a set of classical mathematical riddles with wording that would promote a wrong initial intuition (a lure, as described in Attali and Bar-Hillel, 2020). In order to further investigate the underlying mechanism of CRT resolution, meditators, and non-meditators should be compared on both the CRT and classical mathematical riddles. It would be interesting to see if this effect generalizes to other paradigms that also illustrate a divergence between the incorrect intuitive response and the correct deliberate response. For example, in other mathematical problems that rely on the representation of contextual information, (i.e., the pigeonhole principle; Jacquet and Baratgin, 2023), or different attentional factors, (i.e., causal reasoning; Hattori I. et al., 2017; Hattori M. et al., 2017), or again on the representation of a problem's situation, (i.e., belief revision situation; Baratgin, 2009, 2015; Baratgin and Politzer, 2010; Baratgin et al., 2017; Bar-Hillel et al., 2018). It would also be interesting to compare the influence of activities similar to mindfulness (Sonnier et al., 2023) with that of mindfulness itself on problem-solving. In the present study, mindfulness is practiced with secular intent. However, given that epistemically suspect beliefs (i.e. religious beliefs, romantic beliefs, alternative medicines, etc.) are predicted by cognitive style (intuitive or analytical; Trémolière and Djeriouat, 2019), it would be interesting to replicate this study with people practicing mindfulness with religious intent. Our study reinforces and highlights the benefits of mindfulness in reasoning tasks, in particular its ability to encourage participants to take a step back and automate the de-automatization of information processing.

## Data availability statement

The datasets presented in this study can be found in online repositories. The names of the repository/repositories and accession number(s) can be found at: https://osf.io/x6zas/?view_only=a7b63dd27c744c1b9defcabaff73eab5.

## Ethics statement

The studies involving humans were approved by Ethics Committee of P-A-R-I-S Association. The studies were conducted in accordance with the local legislation and institutional requirements. The participants provided their written informed consent to participate in this study.

## Author contributions

LL: Conceptualization, Data curation, Investigation, Methodology, Writing—original draft, Writing—review and editing. BJ: Formal analysis, Methodology, Visualization, Writing—review and editing. MB: Writing—review and editing. JB: Conceptualization, Methodology, Supervision, Validation, Writing—review and editing.

## Footnotes

^1^For exercise details, see https://osf.io/x6zas/?view_only=a7b63dd27c744c1b9defcabaff73eab5.

^2^*Fringe consciousness* refers to representations that are not accessible to consciousness but are relevant to focal awareness, such as experiences on the tip of the tongue, feelings of rightness and wrongness, of familiarity and novelty, or of knowledge (Mangan, 2001).

^3^A figure explaining the two rounds is available at: https://osf.io/x6zas/?view_only=a7b63dd27c744c1b9defcabaff73eab5.

^4^The Ethics Committee declaration and the consent form are available at: https://osf.io/x6zas/?view_only=a7b63dd27c744c1b9defcabaff73eab5.

^5^The data, R code for the analyses, and supplementary tables are available on the online repository available at: https://osf.io/x6zas/?view_only=a7b63dd27c744c1b9defcabaff73eab5.

^6^Tables containing the summary of the confidence analyses for each riddle are available on the online repository at: https://osf.io/x6zas/?view_only=a7b63dd27c744c1b9defcabaff73eab5.
